# Supplementary material for: Effect of Hen Genotype and Laying Time on Egg Quality and Albumen Lysozyme Content and Activity
Source: Animals (Basel). 2023 May 11;13(10):1611. doi: 10.3390/ani13101611 (PMC10215720; doi:10.3390/ani13101611)
Supplement: Supplementary file 1 [file animals-13-01611-s001.zip › animals-2274060-supplementary.pdf]

Table S1. Correlations between some egg quality traits and lysozyme content and activity in the eggs of 56-week-old hens of 4 conservation strains collected at different times of the day

**Item**	**Lysozyme content (%)**				**Lysozyme activity (U/ml)**			
	**thin albumen**		**thick albumen**		**thin albumen**		**thick albumen**	
	**Time of egg collection (h)**							
	**7:00**	**13:00**	**7:00**	**13:00**	**7:00**	**13:00**	**7:00**	**13:00**
Egg weight (g)	-0.237	-0.379$^{\Delta}$	-0.387$^{\Delta}$	-0.558$^{\Delta}$	0.224	0.389$^{\Delta}$	-0.087	-0.170
Air cell height	0.162	0.433$^{\Delta}$	-0.073	0.358$^{\Delta}$	0.454$^{\Delta}$	0.100	0.105	0.237
Albumen height (mm)	-0.718$^{\Delta}$	-0.657$^{\Delta}$	-0.397$^{\Delta}$	-0.834$^{\Delta}$	0.214	0.500$^{\Delta}$	-0.094$^{\Delta}$	-0.277$^{\Delta}$
Haugh unit	-0.708$^{\Delta}$	-0.610$^{\Delta}$	-0.338$^{\Delta}$	-0.791$^{\Delta}$	0.176	0.493$^{\Delta}$	-0.082	-0.269$^{\Delta}$
Albumen pH	0.613$^{\Delta}$	0.482$^{\Delta}$	0.475$^{\Delta}$	0.792$^{\Delta}$	0.051	-0.551$^{\Delta}$	0.221^{x}	0.260$^{\Delta}$
Yolk weight (g)	0.028	-0.146	0.005	-0.381$^{\Delta}$	0.372$^{\Delta}$	0.409$^{\Delta}$	-0.117	-0.006
Yolk pH	0.364$^{\Delta}$	0.191	0.364$^{\Delta}$	0.416$^{\Delta}$	-0.486$^{\Delta}$	-0.598$^{\Delta}$	-0.146	-0.002
Albumen weight (g)	-0.257$^{\Delta}$	-0.258$^{\Delta}$	-0.426$^{\Delta}$	-0.428$^{\Delta}$	0.072	0.223	-0.062	-0.164
Yolk colour: L*	-0.138	0.062	-0.088	-0.030	-0.201	0.039	-0.054	-0.015
a*	0.030	-0.158	-0.113	-0.205	0.196	0.074	0.183	-0.070
b*	-0.254	-0.362$^{\Delta}$	-0.334$^{\Delta}$	-0.395$^{\Delta}$	0.024	0.184	0.071	-0.129
Shell colour: L*	-0.036	-0.233	-0.406$^{\Delta}$	0.123	-0.182	-0.153	0.146	0.240
a*	-0.060	0.129	0.309$^{\Delta}$	-0.256	0.295$^{\Delta}$	0.218	-0.183	-0.301$^{\Delta}$
b*	0.340$^{\Delta}$	0.469$^{\Delta}$	0.590$^{\Delta}$	0.162	0.060	-0.035	-0.095	-0.081
Shell weight (g)	-0.318$^{\Delta}$	-0.413$^{\Delta}$	-0.383$^{\Delta}$	-0.499$^{\Delta}$	0.250	0.273^{y}	0.024	-0.127
Shell thickness (**μm**)	-0.330$^{\Delta}$	-0.350$^{\Delta}$	-0.298$^{\Delta}$	-0.261$^{\Delta}$	0.230	0.059	0.031	0.050
Shell porosity (pores/cm^2)	-0.699$^{\Delta}$	-0.475$^{\Delta}$	-0.406$^{\Delta}$	-0.480$^{\Delta}$	0.144	0.001	-0.177	-0.099
Shell crushing strength (N)	-0.225	-0.287$^{\Delta}$	-0.010	-0.359$^{\Delta}$	0.248	0.157	0.186^{x}	-0.273$^{\Delta}$

Columns with the superscript (Δ) are significantly correlated ($p < 0.05$) for time of egg collection and each trait; L* – lightness, a* – redness, b* – yellowness
